# Screening and Carrier Rate of Neuronal Ceroid Lipofuscinosis in Chihuahua Dogs in Japan

**DOI:** 10.3390/ani12091210

**Published:** 2022-05-07

**Authors:** Shahnaj Pervin, Md Shafiqul Islam, Naomi Tada, Toshihiko Tsutsui, Mohammad Mahbubur Rahman, Akira Yabuki, Martia Rani Tacharina, Tofazzal Md Rakib, Shinichiro Maki, Osamu Yamato

**Affiliations:** 1 Laboratory of Clinical Pathology, Joint Faculty of Veterinary Medicine, Kagoshima University, 1-21-24 Korimoto, Kagoshima 890-0065, Japan; s.pervin30@yahoo.com (S.P.); si.mamun@ymail.com (M.S.I.); tada-n@bioart.or.jp (N.T.); mahbub57991@yahoo.com (M.M.R.); yabu@vet.kagoshima-u.ac.jp (A.Y.); martia.rt@fkh.unair.ac.id (M.R.T.); rakibtofazzal367@gmail.com (T.M.R.); k6993382@kadai.jp (S.M.); 2 Department of Pathology and Parasitology, Faculty of Veterinary Medicine, Chattogram Veterinary and Animal Sciences University, Khulshi, Chattogram 4225, Bangladesh; 3 Japan Institute of Small Animal Reproduction (Bio Art), 3-16-9 Uchikanda, Chiyoda-ku, Tokyo 101-0047, Japan; tsutsui-t@bioart.or.jp; 4 Faculty of Veterinary Medicine, Airlangga University, Campus C, Jl. Mulyorejo, Surabaya 60115, Indonesia

**Keywords:** carrier rate, Chihuahua dog, *CLN7*/*MFSD8* gene, neuronal ceroid lipofuscinosis, real-time PCR assay

## Abstract

**Simple Summary:**

Neuronal ceroid lipofuscinosis (NCL) is a rare group of lethal neurodegenerative lysosomal storage diseases, in which a homozygous single base-pair deletion (c.846delT) in the canine *CLN7*/*MFSD8* gene has been identified as a causative mutation in Chihuahuas. In this study, we aimed to determine the carrier rate of NCL in Chihuahuas in Japan based on the screening of puppies using a newly designed real-time PCR assay. A survey conducted among 1007 Chihuahua puppies indicated that the carrier rate was 1.29%. Given the lethality of this disease, the mutant allele frequency (0.00645) is sufficiently high to warrant measures for disease control and prevention using the genotyping assay.

**Abstract:**

Neuronal ceroid lipofuscinosis (NCL) is a group of rare lethal neurodegenerative lysosomal storage diseases that occur in a range of dog breeds, including Chihuahuas. Recently, a homozygous single base-pair deletion (c.846delT), which causes a frame shift generating a premature stop codon (p.Phe282Leufs13*) in the canine *CLN7*/*MFSD8* gene, has been identified as a causative mutation for NCL in Chihuahuas. The objective of this study was to determine the frequency of the mutant allele and/or carrier rate of NCL in Chihuahuas in Japan using a newly designed real-time PCR assay. Samples of saliva were randomly collected from 1007 Chihuahua puppies during physical examinations prior to the transportation to pet shops. Screening results revealed a carrier rate of 1.29%, indicating a mutant allele frequency (0.00645) that is considered sufficiently high to warrant measures for the control and prevention of this lethal disease. The genotyping assay designed in this study could make a valuable contribution to the control and prevention of NCL.

## 1. Introduction

Neuronal ceroid lipofuscinosis (NCL) is a recessively inherited progressive neurodegenerative lysosomal storage disorder detected in both humans and non-human animals, which is characterized by the abnormal accumulation of autofluorescent lysosomal storage bodies in the central nervous system, retina, and other tissues throughout the body [1,2,3]. NCL has been described in many species, including cattle, sheep, pigs, cats, mice, non-human primates, and birds, and most frequently in different dog breeds, including Chihuahuas [4,5]. When the disease occurs in humans and dogs, it has similar clinical manifestations, including behavioral abnormalities, visual complications, brain atrophy, seizures, and motor disfunctions, and eventually leads to premature death [2,3].

To date, NCL has been described in numerous different dog breeds, including English Setters [6], Dachshunds [7,8], Chinese Crested Dogs [9], Salukis [10], Labrador Retrievers [11], Border Collies [12,13,14], Cocker Spaniels [15], American Bulldogs [16], Australian Shepherds [17,18], Chihuahuas [19,20], Australian Cattle Dogs [21,22], Tibetan Terriers [23,24,25,26], Polish Owczarek Nizinny Dogs [26], American Staffordshire Terriers [27], Cane Corso Dogs [28], Alpenländische Dachsbracke Dogs [29], German Shorthaired Pointers [30], Golden Retrievers [31], Welsh Corgis [3], Yugoslavian Shepherds [32], Dalmatians [33], Shikoku Dogs [34], and three types of mixed breed dogs (Australian Cattle Dog-Australian Shepherd mix [35], German Shepherd-Australian Cattle Dog mix, and a dog with unknown ancestry, although it appeared to be a Beagle and Labrador Retriever mix [36]). Among these breeds, the following nine canine orthologs of the human NCL-associated genes have been identified as causal genes in canine NCL: *CLN1*/*PPT1* [8,28], *CLN2*/*TPP1* [7,26], *CLN5* [12,13,14,21,31], *CLN6* [17,18], *CLN7*/*MFSD8* [9,20], *CLN8* [29,30], *CLN10*/*CTSD* [16], *CLN12*/*ATP13A2* [22,23,24,25], and *ARSG* [27].

The occurrence of NCL in Chihuahuas was first reported in Australia and New Zealand in the 1970s [37,38], and has subsequently been reported in Japan in 2003 [39] and 2011 [19]. Recently, a homozygous single base-pair deletion (c.846delT; g.1301076delT, CanFam3.1) in exon 8 in the canine *CLN7*/*MFSD8* gene, which causes a frame shift and thereby generates a premature stop codon (p.Phe282Leufs13*), has been identified in two littermates from Scotland [40] and in four dogs from Japan, Italy, and England in 2016 [20]. In the same year, an affected Chihuahua was also reported to be molecularly diagnosed with this mutation in Switzerland [41], whereas in 2015, the mutation had been identified for the first time in a Chinese Crested Dog with NCL in the USA [9]. The *CLN7*/*MFSD8* gene encodes the putative lysosomal transporter that is relevant for lysosomal motility and plays an important role for neuronal cell survival under conditions of starvation [42]. Loss of this transporter may result in enhanced neuronal death through dysfunctions of late endosomes and lysosomes.

In accordance with the Japan Kennel Club (JKC: https://www.jkc.or.jp (accessed on 27 April 2022)), certified by the Federation Cynologique Internationale (FCI: http://www.fci.be/ (accessed on 27 April 2022)), from 2001 to the present (2021), Chihuahuas have been the second most commonly registered dog breed in Japan. During this period, an average of 64,200 Chihuahua puppies were registered annually by the JKC. The presence of fatal inherited diseases, such as NCL, in this popular breed is a serious problem if the frequency of the mutant allele is sufficiently high to sporadically give rise to affected dogs.

To the best of our knowledge, neither the mutant allele frequency nor the carrier rate of NCL have been determined for Chihuahuas in Japan. Accordingly, we believe it to be of particular importance to establish the genetic status of NCL in Japanese Chihuahuas, which would enable the supply of healthy dogs to breeders and pet owners, as well as indicate the necessity to take preventive measures to eradicate this devastating neurological disorder. In this regard, a simple and reliable genotyping method is deemed necessary for the large-scale screening of dogs. To meet this need, we sought in this study to develop a real-time PCR assay that could be used to determine the mutant allele frequency and/or carrier rate of NCL in Chihuahuas in Japan.

## 2. Materials and Methods

The experiments conducted in this study were performed in accordance with the guidelines regulating animal use and ethics at Kagoshima University (No. VM15041; approval date: 29 September 2015), and informed consent was obtained from cooperating breeders.

### 2.1. Sample Collection and Storage

Using sterile cotton swabs, saliva samples were randomly collected from 1007 clinically healthy Chihuahua puppies at the time of medical checks performed by an animal technician (N.T.) and a veterinarian (T.T.) at a corporate animal hospital (Bio Art) in Tokyo, Japan, with the informed consent of the breeders who owned these puppies. These samples were spotted onto Flinders Technology Associates (FTA) filter papers (FTA Classic Card; Whatman International Ltd., Piscataway, NJ, USA) and stored in a refrigerator (4 °C) until used for the extraction of DNA. DNA samples from two healthy dogs for the homozygous wild-type allele, two heterozygous carriers, and 12 affected dogs for the homozygous mutant allele, which had been determined in a previous study [19,20], were used to evaluate the results of the genotyping assay. In addition, these genotypes have been officially confirmed by Sanger sequencing (Kazusa Genome Technologies Ltd., Kisarazu, Japan) using the specific primers listed in Table 1.

### 2.2. Genotyping of the c.846delT Mutation in the Canine CLN7/MFSD8 Gene

The primers and TaqMan minor groove binder (MGB) probes used for the real-time PCR assay (the sequences of which are listed in Table 1) were designed based on the sequences of the canine *CLN7*/*MFSD8* gene in wild-type dogs (GenBank Accession No. NC_006601.3). These primers and probes, each of which was linked to a fluorescent reporter dye (6-carboxyrhodamine or 6-carboxyfluorescein) at the 5′-end and a non-fluorescent quencher dye at the 3′-end, were synthesized by a commercial company (Applied Biosystems, Foster City, CA, USA). For the preparation of DNA templates, we used a disc punched out of the FTA cards for DNA extraction, as previously described [13]. Real-time PCR amplifications were carried out in a final volume of 5 µL consisting of a 2× PCR master mix (TaqMan GTXpress Master Mix, Applied Biosystems), 80× genotyping assay mix (TaqMan SNP Genotyping Assays, Applied Biosystems) containing the specific primers and TaqMan MGB probes, and template DNA. A negative control containing nuclease-free water rather than DNA was included in each run. The cycling conditions consisted of 20 s at 95 °C, followed by 50 cycles of 3 s at 95 °C and 20 s at 60 °C, with a subsequent holding stage at 25 °C for 30 s. The data obtained were analyzed using StepOne version 2.3 (Applied Biosystems). In addition, an allelic discrimination plot was constructed based on the three types of amplification plots (homozygous wild-type, heterozygous, and homozygous mutant). These data were calculated using software based on the results obtained using the reference DNA samples from 45 dogs (20 homozygous wild-type, 13 heterozygous carrier, and 12 homozygous mutant).

### 2.3. Statistical Analysis

The allele frequencies obtained in this study were analyzed using Chi-square tests for Hardy–Weinberg equilibrium. Deviations between the measured and expected values were considered statistically significant at *p* < 0.05.

## 3. Results

**Figure 1:**
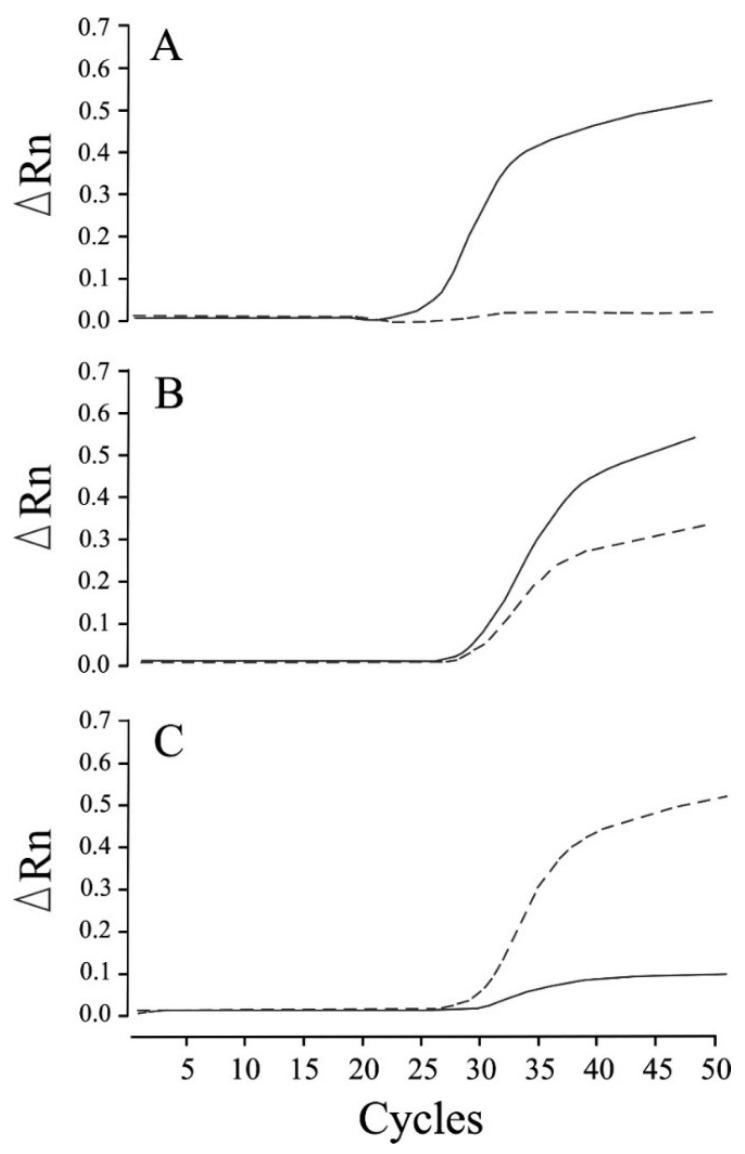
Real-time PCR amplification plots of wild-type and mutant alleles for neuronal ceroid lipofuscinosis in Chihuahuas. Amplification was plotted as fluorescence intensity (ΔRn values) against the cycle number. ΔRn values represent the reporter dye signal normalized to the internal reference dye and corrected for the baseline signal established in the initial few PCR cycles. The three amplification plots show the homozygous wild-type (**A**), heterozygous carrier (**B**), and homozygous mutant (affected) (**C**) genotypes. The solid and dotted lines indicate amplification in the presence of wild-type and mutant alleles, respectively.

**Figure 2:**
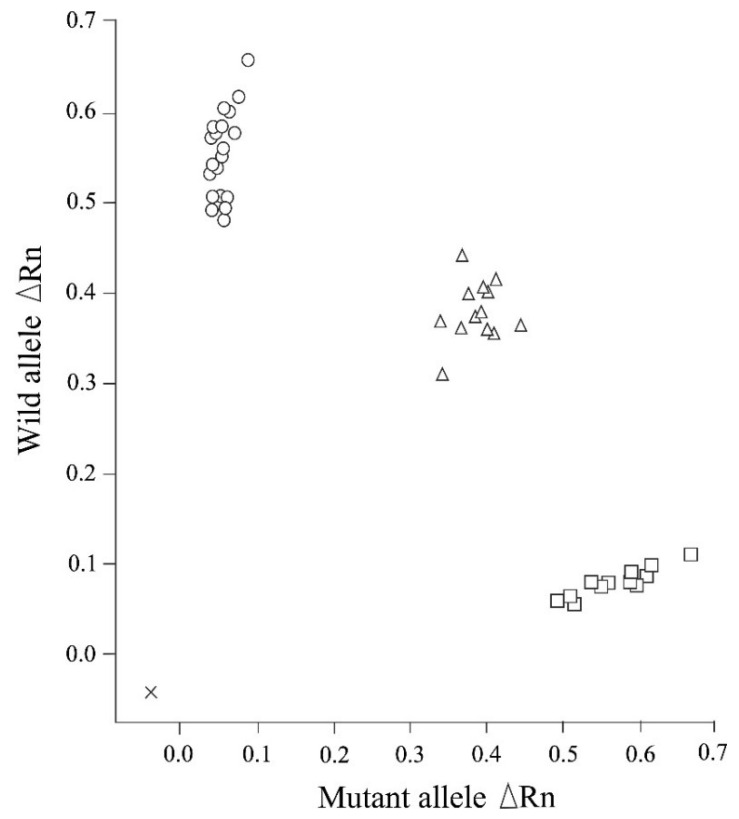
An allelic discrimination plot of end point fluorescence real-time PCR data showing the three genotypes (homozygous wild-type, heterozygous carrier, and homozygous mutant) of neuronal ceroid lipofuscinosis in Chihuahuas. This plot presents the data obtained for 45 representative DNA samples of Chihuahuas that had been previously genotyped. The plot shows the fluorescence intensities (ΔRn values) for each allele. ×: No template control; ○: Homozygous wild-type genotype (20 samples); ∆: Heterozygous carrier genotype (13 samples); □: Homozygous mutant (affected) genotype (12 samples).

We established that the newly designed real-time PCR assay assessed in this study can be used to clearly distinguish genotypes related to NCL in Chihuahuas after 50 cycles of amplification, in the absence of any non-specific allelic amplification (Figure 1). The real-time PCR genotyping results obtained for the control samples were found to be consistent with those obtained based on Sanger sequencing. Moreover, this assay enabled us to construct allelic discrimination plots based on the amplification plots obtained for the three genotypes using the DNA samples obtained from 45 dogs (20 homozygous wild-type, 13 heterozygous carrier, and 12 affected dogs) (Figure 2).

Genotyping based on the screening of puppies revealed that among the surveyed population of 1007 Chihuahuas, there were 994 homozygous wild-type dogs, 13 heterozygous carriers, and no affected dogs. On the basis of these observations, we estimated a carrier rate of 1.29%. The corresponding mutant allele frequency was 0.00645, indicating that the expected frequencies of homozygous wild-type, heterozygous carrier, and homozygous mutant genotypes were 0.987, 0.0128, and 0.0000417, respectively. Chi-square test analysis (χ2 = 0.042503, df = 2, *p*-value = 0.979) indicated that these three genotypes were in Hardy–Weinberg equilibrium.

## 4. Discussion

The clinical signs and history of NCL in Chihuahuas are particularly severe and devastating [3,19,39]. The onset of neurological signs is typically detected between 13 and 21 months of age, and these grow in severity with increasing age. Requests from owners for euthanasia or sudden death attributable to status epilepticus generally occur at 13 to 24 months of age. The signs include visual defect, a startled response to sound and touch, ataxia, and epileptic seizure [3,19,36,37,39]. Long-term nursing care for affected dogs with these progressive neurological signs can represent a considerable mental burden for their owners, and consequently, even a few cases of NCL among dogs could have a substantial impact on their owners and breeders.

In the present study, we established that the carrier rate and mutant allele frequency of NCL in the Chihuahua study population were 1.29% and 0.00645, respectively. On the basis of this frequency, the expected number of heterozygous carriers and affected dogs among the mean number of Chihuahua puppies (approximately 64,200/year) registered in recent years are 8234 and 2.67, respectively, thereby indicating that approximately 8000 carriers and 2 to 3 affected dogs might be bred annually in Japan. Consistent with these estimates, in the past decade, more than 20 affected dogs were molecularly diagnosed in our laboratory, thereby supporting the diagnosis of this disease in Japan [19,20]. Furthermore, reports of affected dogs in multiple countries, including Australia, New Zealand, Japan, Scotland, Italy, England, and Switzerland [19,20,37,38,39,40,41], provide evidence to indicate that the causative c.846delT mutation is already extensively distributed worldwide. Consequently, this raises concerns regarding the health and breeding ethics of Chihuahuas, one of the most popular canine breeds, not only in Japan but also worldwide. Accordingly, given the established lethality of NCL, the figures obtained in this study for the carrier rate (1.29%) and mutant allele frequency (0.00645) are deemed sufficiently high to warrant prevention measures for the disease control.

Real-time PCR techniques have been widely used for the detection of mutations in genes causing inherited diseases in both humans and non-human animals [43]. A notable advance in this regard has been the development of real-time PCR assays performed in combination with the usage of FTA cards for sample collection, which has eliminated the need for multiple steps of extraction and purification methods, thereby enabling rapid processing and reporting of the results in approximately 2 h after sampling [13]. Moreover, collection of saliva using FTA cards contributes to the reduction of distress caused when handling animals. Accordingly, we believe that the real-time PCR method designed in the present study would make an important contribution in efforts to control and prevent NCL in Chihuahuas.

In the present study, we surveyed puppies directly to determine the carrier rate and mutant allele frequency of NCL in Chihuahuas. Ideally, this screening should be conducted for all dogs bred from kennels to comprehensively establish the risk of producing carriers and affected dogs. However, blanket screening of this nature is considered an ineffective and high-cost approach. In addition, breeders should be notified of the detection of carriers, thereby enabling them to identify parent carrier dogs. Consequently, the comprehensive screening of puppies might not contribute effectively to the prevention and eradication of canine inherited diseases, and with respect to preventing the production of affected dogs and reducing the number of carriers, it would be more productive and less costly to survey only breeding dogs reared by breeders and undertake the appropriate management of mating.

In Japan, genotyping surveys have been previously performed for several canine inherited diseases to determine the associated carrier rates and mutant allele frequencies, and thereby evaluate the necessity for preventive measures. Among these diseases, lethal disorders characterized by progressive neurological disfunctions include GM1 gangliosidosis in Shiba Inus (carrier rate: 1.02%) [44], GM2 gangliosidosis variant 0 (Sandhoff disease) in Toy Poodles (0.20%) [43], and NCL in Border Collies (8.11%) [13]. Similar to Chihuahuas, Shiba Inus and Toy Poodles are also particularly popular canine breeds in Japan, and consequently, underlying lethal diseases in these breeds could be expected to have wide-ranging implications, such as those associated with NCL in Chihuahuas. Indeed, as with NCL in Chihuahuas, Shiba Inus affected with GM1 gangliosidosis have continued to be detected at the rate of 1 to 5 affected dogs per year, not only until 2012 [44], but also up to the present day. In contrast, Toy Poodles affected with Sandhoff disease have not been diagnosed since four affected dogs were reported in 2014 [43], which can probably be attributed to the low mutant allele frequency in the Japanese Toy Poodle population, and thus indicates that urgent preventive measures are currently unnecessary. Furthermore, there has been a high mutant allele frequency and an increasing trend among Border Collies affected with NCL since 2012 [13,14]. However, appropriate remedial measures, including the examination of all breeding dogs, particularly in kennels with high prevalence, and subsequent appropriate breeding control, have contributed to a declining trend [14], with no positive cases reported recently.

Preventive measures should be undertaken in an appropriate manner in full cooperation with involved breeders and nationwide or specific kennel clubs, having initially assessed the current mutant allele frequencies and population numbers of the implicated dog breeds in each region and country. An assessment of the data obtained from systematic and functional monitoring surveys indicates that continuous removal of carriers from breeding colonies might be the most effective measure for preventing and eradicating fatal hereditary diseases in purebred dogs.

## 5. Conclusions

On the basis of the results obtained in this study, we established that in Japan, the carrier rate of NCL in Chihuahuas is currently 1.29%, and given the lethality of this disease, the corresponding mutant allele frequency (0.00645) is deemed sufficiently high to warrant measures for disease control and prevention. Ideally, in this regard, it is considered important to conduct extensive molecular screening of breeding Chihuahuas in JKC-registered kennels throughout Japan. To this end, we believe that the genotyping assay designed in this study will make a potentially valuable contribution to the control and prevention of NCL in Chihuahuas.

## Figures and Tables

**Table 1:** Sequences of the primers and probes used in the real-time PCR assay and Sanger sequencing for the c.846delT mutation in the canine *CLN7*/*MFSD8* gene.

Primer/Probe	Sequence 5′ to 3′ (mer)	Position	Reporter (5′)	Quencher (3′)	Tm (°C)	Concentration (nM)
Real-time PCR:						
Forward primer	GCTGTTGTGGCCACTAATATTGTG (24)	c.805_828	NA	NA	66.1	450
Reverse primer	AGAATAAAACTTACGTTTCAAAAAGGGCAA (30)	c.851_866+14	NA	NA	68.1	450
Probe for wild-type allele	TTCGTGATTCTATTTATCT (19)	c.832_850	FAM	NFQ	48.4	100
Probe for mutant allele	TTTTTCGTGATTCTATTATCT (21)	c.829_850	VIC	NFQ	52.3	100
Sanger sequencing:						
Forward primer	GTCATAGAATTTGCTACATATAATTTC (27)	Intron 7	NA	NA	57.1	NA
Reverse primer	GTTTTGAGAACATTGATATGCTTGAT (26)	Intron 8	NA	NA	62.9	NA

Tm: Melting temperature calculated using Oligo Calculator (http://www.ngrl.co.jp/tools/0217oligocalc.htm (accessed on 27 April 2022)); NA: Not applicable; FAM: 6-Carboxyfluorescein; VIC: 6-Carboxyrhodamine; NFQ: Non-fluorescent quencher.

## Data Availability

Not applicable.
